# Enhancement of anti-STLV-1/HTLV-1 immune responses through multimodal effects of anti-CCR4 antibody

**DOI:** 10.1038/srep27150

**Published:** 2016-06-02

**Authors:** Kenji Sugata, Jun-ichirou Yasunaga, Michi Miura, Hirofumi Akari, Atae Utsunomiya, Kisato Nosaka, Yuko Watanabe, Hitoshi Suzushima, Ki-Ryang Koh, Masanori Nakagawa, Michinori Kohara, Masao Matsuoka

**Affiliations:** 1 Laboratory of Virus Control, Institute for Virus Research, Kyoto University, Kyoto, Japan; 2 Japan Society for the Promotion of Science (JSPS), Chiyoda-ku, Tokyo, Japan; 3 Laboratory of Evolutional Virology, Institute for Virus Research, Kyoto University, Kyoto, Japan; 4 Department of Hematology, Imamura Bun-in Hospital, Kagoshima, Japan; 5 Department of Hematology, Kumamoto University School of Medicine, Kumamoto, Japan; 6 Department of Hematology, Kumamoto Shinto General Hospital, Kumamoto, Japan; 7 Department of Hematology, Osaka General Hospital of West Japan Railway Company, Osaka, Japan; 8 North Medical Center, Kyoto Prefectural University of Medicine, Yosano-cho, Kyoto, Japan; 9 Department of Microbiology and Cell Biology, Tokyo Metropolitan Institute of Medical Science, Tokyo, Japan

## Abstract

Human T-cell leukemia virus type 1 (HTLV-1) causes adult T-cell leukemia and inflammatory diseases. Because anti-HTLV-1 immune responses are critical for suppressing infected cells, enhancing cellular immunity is beneficial for the treatment of HTLV-1-associated diseases. Using simian T-cell leukemia virus type 1 (STLV-1) infected Japanese macaques, we analyzed the immune responses to viral antigens and the dynamics of virus-infected cells. The chemokine receptor CCR4 is expressed on STLV-1 infected cells, and administration of humanized monoclonal antibody to CCR4, mogamulizumab, dramatically decreased the number of STLV-1-infected cells *in vivo*. Concurrently, mogamulizumab treatment enhanced STLV-1 specific CD4^+^ and CD8^+^ T cell responses by simultaneously targeting CCR4^+^ effector regulatory T (Treg) cells and infected cells. Mogamulizumab promoted the phagocytosis of CCR4^+^ infected cells by macrophages, which likely enhanced antigen presentation. Vaccination with recombinant vaccinia virus (rVV) expressing viral antigens suppressed the proviral load and the number of Tax-expressing cells. Enhanced T-cell responses were also observed in some ATL patients who were treated with mogamulizumab. This study shows that mogamulizumab works not only by killing CCR4^+^ infected cells directly, but also by enhancing T cell responses by increasing the phagocytosis of infected cells by antigen-presenting cells and suppressing CCR4^+^ effector Treg cells.

Human T-cell leukemia virus type 1 (HTLV-1) is transmitted mainly via cell-to-cell infection, and the infectivity of cell-free virus is very poor; in this respect it differs from another human retrovirus, human immunodeficiency virus type 1 (HIV-1)^1^,^2^. To enhance its transmission, HTLV-1 causes the proliferation and promotes the survival of infected cells *in vivo*^3^,^4^. The host immune response to this virus has a tremendous impact on the dynamics of the HTLV-1 infected cell population. In particular, the host HLA allele has been shown to be critical for controlling the proliferation of infected cells^5^. Tax is an immunodominant viral protein, and cytotoxic T-lymphocytes (CTLs) to Tax influence the dynamics of infected cells^6^. Furthermore, the cellular immune response to HTLV-1 bZIP factor (HBZ) is also critical in determining the proviral load in HTLV-1 infected individuals^7^. Recently, we reported that CTLs to HBZ have protective effects against adult T-cell leukemia (ATL)^8^.

C-C chemokine receptor type 4 (CCR4) is expressed on T helper type 2 (Th2) cells, regulatory T (Treg) cells and skin-homing T cells. Its ligands, CCL-17 and −22, are expressed in the lung, skin, intestine and liver^9^. Treg cells have been reported to utilize CCR4 for peripheral migration in mice^10^. Recent studies reveal that the immunophenotype of HTLV-1 infected cells is CD4^+^CCR4^+^CADM1^+ ^11,12, indicating that this virus targets a specific subpopulation of T cells *in vivo*. ATL cells are also CD4^+^CCR4^+^CADM1^+^, suggesting that HTLV-1 infects or increases this subpopulation of T cells and finally transforms them^13^. Recently, humanized anti-CCR4 monoclonal antibody (mAb), mogamulizumab, has been developed for the treatment of ATL^14^. CCR4 is a marker for effector Treg cells in humans, and depletion of CCR4^+^ Treg cells *in vitro* causes the enhancement of anti-tumor immunity^15^. Therefore, depletion of Treg cells by CCR4 mAb in humans might also be beneficial in vaccine and cancer therapy in general^16^.

In this study, we found that mogamulizumab treatment induced a long-lasting decrease in the number of simian T-cell leukemia virus type 1 (STLV-1) infected cells *in vivo* by enhancing T-cell responses to viral antigens and suppressing Treg cells *in vivo*. Similarly, anti-Tax and anti-HBZ T cells were increased in some ATL patients who received mogamulizumab treatment. Thus, mogamulizumab elicits anti-HTLV-1 effects by killing CCR4^+^ T cells and enhancing T-cell responses to HTLV-1.

## Results

### Mogamulizumab treatment induces long-term suppression of the STLV-1 proviral load

**Figure 1:**
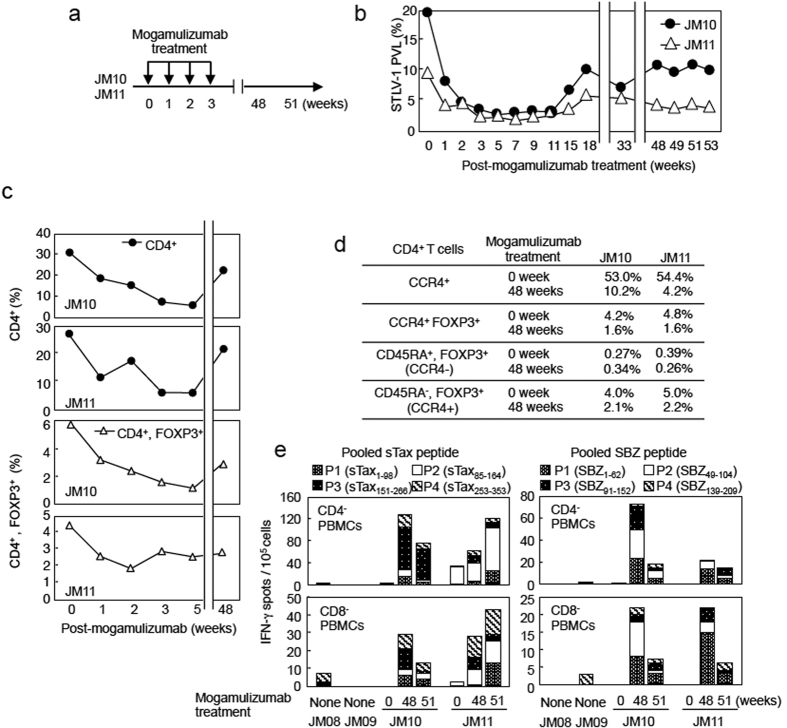
Mogamulizumab induces the activation of a virus-specific T-cell response in STLV-1-infected Japanese macaques. (**a**) Scheme of mogamulizumab treatment in JM10 and JM11. (**b**) Changes in proviral load (PVL) in STLV-1-infected JM10 and JM11 after mogamulizumab treatment. PVL data before 18 weeks were reported previously^17^. (**c**) Changes in percent of PBMCs that are CD4^+^ or CD4^+^FOXP3^+^ after mogamulizumab treatment. (**d**) Proportion of CD4^+^ cells that are CCR4^+^, CCR4^+^FOXP3^+^, CD45RA^+^FOXP3^+^ or CD45RA^−^FOXP3^+^ at pre- and post- mogamulizumab treatment. Positive cells were gated based on each isotype control (>0.2%). (**e**) T-cell responses to sTax and SBZ before and after treatment with mogamulizumab. Monkey PBMCs were obtained before (0 weeks) and after (48 and 51 weeks) treatment and their response was measured by IFN-γ ELISPOT assay using pooled peptides of sTax or SBZ. Data from STLV-1-infected JM08/09 (before mogamulizumab treatment and vaccination) are presented as controls.

We have previously described STLV-1 naturally infected Japanese macaques (*Macaca fuscata*) (JMs) as a model of HTLV-1 infection, and we reported that mogamulizumab strongly suppressed the number of STLV-1-infected cells *in vivo*^17^. We now report that after administration of mogamulizumab, the proviral load rebounded to less than half of its original level even after 53 weeks (Fig. 1a,b) (JM10 from 19.10% to 9.51%: JM11 from 8.95% to 3.35%). The half-life of this antibody is approximately 18.2 days in humans^14^, suggesting that at 53 weeks, the proviral load was suppressed by a mechanism other than direct killing of infected cells by mogamulizumab. Since it has been reported that Treg cells express CCR4 and suppress the protective immune responses to cancer and pathogens^15^, we measured the number of Treg cells before and after mogamulizumab treatment. As shown in Fig. 1c, the CD4^+^FOXP3^+^ cell population decreased immediately after mogamulizumab treatment and remained at about 50% of the original level even after 48 weeks. On the other hand, the total CD4^+^ T cell population had substantially recovered at 48 weeks (72% in JM10 and 77% in JM11 compared with 0 week). Among the FOXP3^+^ population at 48 weeks, the number of effector-type Treg cells (CCR4^+^ or CD45RA^−^ CD4^+^ T cells) was dramatically decreased. However, the number of naïve Treg cells (CD45RA^+^FOXP3^+^CD4^+^ T cells) remained unchanged (Fig. 1d).

### Effect of mogamulizumab on the host immune response

We next asked whether mogamulizumab treatment enhances the specific immune response to STLV-1 *in vivo*. Therefore we examined the immune responses to STLV-1 Tax (sTax) and STLV-1 bZIP factor (SBZ), viral antigens which resemble Tax and HBZ of HTLV-1. Indeed, CD4^+^ and CD8^+^ T-cell responses to both sTax and SBZ were remarkably enhanced in both JMs at 48 and 51 weeks post-treatment compared with non-treated JMs, suggesting that the decrease in proviral load was due to enhanced STLV-1 specific immune responses (Fig. 1e). In the ELISPOT assay, we did not observed IFN-γ production of T cells when the monkey PBMCs were stimulated with negative control peptides (H-2^b^-restricted epitope) (Supplementary Fig. S1).

**Figure 2:**
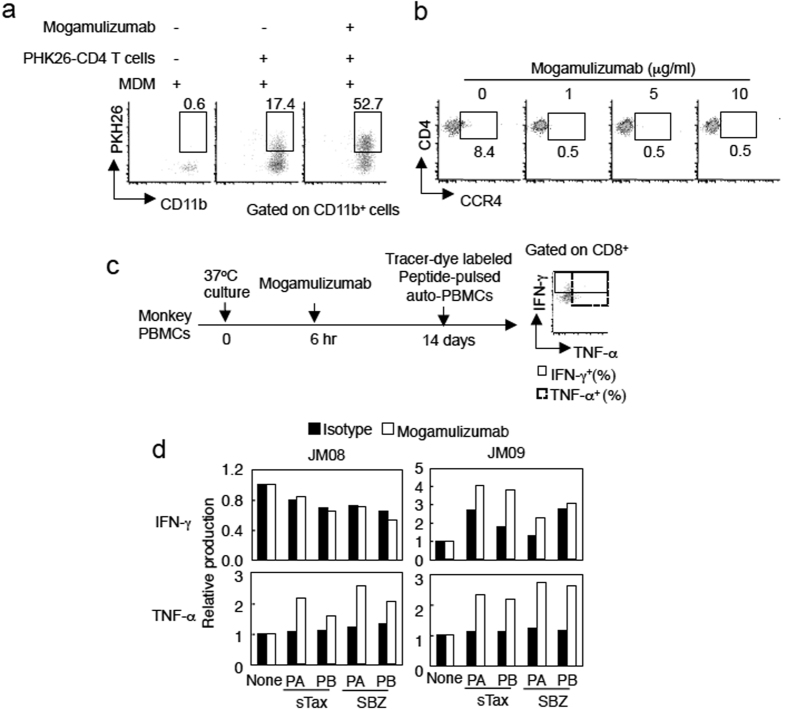
Immune responses induced by mogamulizumab. (**a**) Antibody-dependent phagocytosis by macrophages. PKH26-labeled monkey CD4^+^ T cells from infected JMs were treated with mogamulizumab and co-cultured with monocyte-derived macrophages (MDM), and CD11b^+^PHK26^+^ cells were measured. (**b**) Mogamulizumab depletes CCR4^+^CD4^+^ T cells from PBMCs of an STLV-1 infected monkey *in vitro*. (**c**) Mogamulizumab induces cytokine production in STLV-1 specific CD8 T cells in *ex vivo* culture. PBMCs from unvaccinated STLV-1 infected monkeys were cultured with mogamulizumab, and then stimulated with auto-PBMCs that were pulsed with sTax (PA: sTax_1–164_, PB: sTax_151–353_) or SBZ (PA: SBZ_1–104_, PB: SBZ_91–206_) peptides and labeled with tracer-dye. (**d**) IFN-γ and TNF-α production were measured in the tracer-dye negative population. The data from non-pulsed PBMCs is shown as a reference.

Monoclonal antibody induces not only antibody dependent cellular cytotoxicity (ADCC) but also antibody-dependent phagocytosis against cancer cell *in vivo*. To further clarify how mogamulizumab activates immune responses to STLV-1, we analyzed mogamulizumab dependent T-cell engulfment by phagocytes. PHK26 dye-labeled CD4^+^ T cells from STLV-1 infected JMs were first treated with mogamulizumab and then incubated with monocyte-derived macrophages (MDM). Thereafter, macrophages were stained with anti-CD11b antibody. When target cells were treated with mogamulizumab, phagocytosis increased significantly: CD11b^+^PKH26^+^ cells (i.e., macrophages that had engulfed the labeled CD4^+^ T cells) were increased (to 52.7% of CD11b^+^ cells) compared with the untreated control (17.4%) (Fig. 2a). This suggests that mogamulizumab promotes the phagocytosis of CD4^+^ T cells by antigen-presenting cells, which would lead to augmented CTL responses to sTax and SBZ.

To analyze whether mogamulizumab has similar effects on CD4^+^ T cells *in vitro*, we cultured monkey PBMCs in the presence of the antibody. As shown in Fig. 2b, mogamulizumab strongly reduced the number of CD4^+^ CCR4^+^ T cells *in vitro*. Furthermore, when PBMCs from two STLV-1-infected JMs (JM08 and JM09) were cultured in the presence of mogamulizumab (Fig. 2c), CD8^+^ T cells stimulated with sTax and SBZ pooled peptides produced higher levels of TNF-α (Fig. 2d). In cells from one of these monkeys (JM09), enhanced IFN-γ production was also observed. These results suggest that mogamulizumab elicits T-cell responses to sTax and SBZ through cross-presentation by antigen presenting cells (APCs), likely via ADCC and Fc receptor-mediated phagocytosis *in vitro*.

### Vaccination against sTax and SBZ induces an anti-STLV T-cell response

These data suggest that enhanced memory T cell responses could suppress proviral load *in vivo* after mogamulizumab treatment. To confirm the anti-STLV-1 effect of T cells *in vivo*, we generated recombinant vaccinia viruses (rVVs) expressing mutated sTax and SBZ (Supplementary Fig. S2). Expression levels in 293FT cells were comparable between the wild type and mutant versions (Supplementary Fig. S2a,b). sTax M22 (T130A, L131S), like Tax M22, has lost its NF-κB activating ability (Supplementary Fig. S2c), and SBZ LL/AA (L27A, L28A) has a partially impaired ability to suppress Tax-induced transcription due to missense mutations in its LXXL-like motif (Supplementary Fig. S2d). To check whether these rVVs can induce immune responses, we analyzed IFN-γ producing cells in vaccinated mice. rVVs expressing sTax M22 or SBZ-LL/AA could induce T-cell responses in mice (Supplementary Fig. S2e–j).

**Figure 3:**
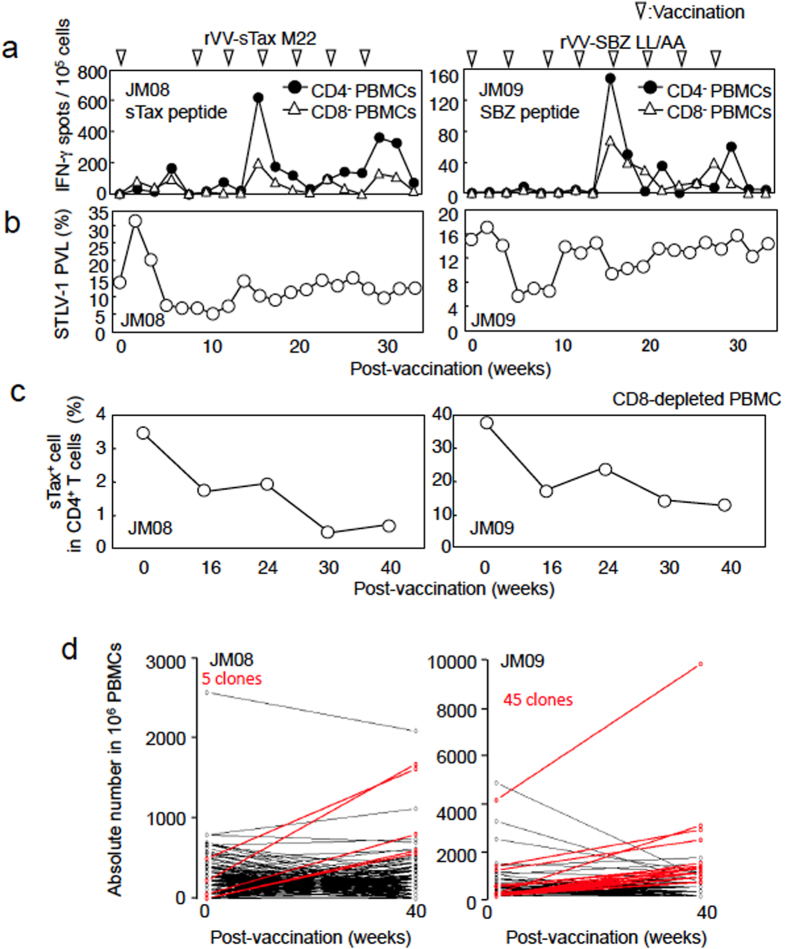
Recombinant vaccinia viruses expressing sTax and SBZ induce immune responses and suppress sTax-expressing cells. (**a**) STLV-1-infected JM08 and JM09 were vaccinated with rVV-sTax M22 or rVV-SBZ LL/AA, respectively. CD4- or CD8- depleted PBMCs were analyzed by IFN-γ ELISPOT assay using sTax or SBZ pooled peptides. Data shown are the total number of spots from pooled peptides (P1–P4) stimulation. (**b**) STLV-1 proviral load in the vaccinated monkeys. (**c**) Decrease in the number of sTax-expressing cells after rVV vaccination. PBMCs from monkeys were depleted of CD8^+^ T cells and cultured for 24 hours, and sTax expressing CD4^+^ T cells were detected. (**d**) High throughput sequencing of STLV-1 integration sites in infected JMs before (0 weeks) and after (40 weeks) vaccination. The number of cells of each STLV-1 clone per 1,000,000 PBMCs was calculated. STLV-1 infected clones that grew more populous and had more than 500 copies at 40 weeks are shown as red lines.

Using rVV expressing sTax M22 or SBZ-LL/AA, we immunized STLV-1 infected JMs (JM08 and JM09, respectively) to analyze the effect of immunization on T-cell responses and proviral load. In JM08, which was immunized with rVV expressing sTax, sTax specific T cells were rapidly detected two weeks after the first vaccination, whereas SBZ specific T-cell responses were detected in JM09 only after four vaccinations, suggesting that the immunogenicity of SBZ is lower than that of sTax (Fig. 3a). Until ten weeks after immunization, proviral load was decreased in both monkeys as shown in Fig. 3b. Thereafter, proviral load gradually recovered in both monkeys despite continued vaccinations. In the sTax-immunized monkey (JM08), sTax expressing cells were suppressed after immunization (3.5% to 0.7%) (Fig. 3c). Likewise, sTax^+^ CD4^+^ T cells were also decreased (38.0% to 13.1%) in the SBZ immunized monkey (JM09), suggesting that CTLs against SBZ also suppress sTax expressing cells *in vivo*. However, even though sTax expression remained suppressed in JM09, proviral load recovered – an observation, which suggests that STLV-1 infected clones lacking sTax expression proliferated or otherwise increased (Fig. 3c). These findings illustrate the heterogeneity of STLV-1 infected cells with regard to sTax expression.

To study the dynamics of STLV-1 infected cells, we analyzed the clonality of STLV-1 infected cells using high throughput sequencing as reported previously to determine the integration sites of the provirus^17^,^18^. As shown in Fig. 3d, most STLV-1 infected clones decreased or did not change in absolute number of cells. However, some clones (red lines) increased after immunization, suggesting that immunization may favor certain STLV-1-infected clones, possibly those with low or absent expression of sTax.

**Figure 4:**
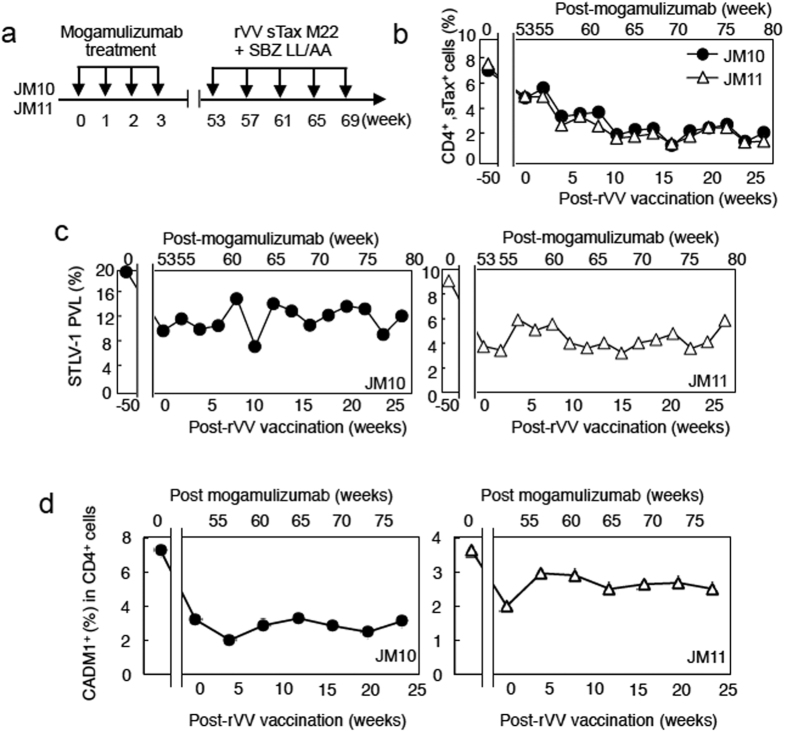
Combined mogamulizumab and rVV vaccination therapy. (**a**) Combined vaccination with rVVs expressing sTax and SBZ after mogamulizumab treatment reduced sTax-expressing cells *in vivo*. The mogamulizumab-treated monkeys shown in Fig. 1a were additionally vaccinated with both rVVs according to the scheme shown. (**b**) The number of sTax-expressing cells in CD8-depleted PBMCs was determined. STLV-1 proviral load (**c**) and CADM1^+^ cells (**d**) in CD4^+^ cells of JM10 and JM11 after vaccination.

T-cell responses to sTax and SBZ were enhanced in mogamulizumab-treated JMs (JM10 and JM11) as shown in Fig. 1e. Therefore, we studied the effect of combined vaccination (sTax M22 plus SBZ LL/AA) on these mogamulizumab-treated monkeys (Fig. 4a). This protocol (treatment with mogamulizumab followed by combined vaccination) did not appear to be more effective than vaccination alone in inducing a T-cell response to STLV-1 (compare Figs 3c and 4b). However, it was interesting that sTax expressing cells were suppressed in both monkeys after vaccination (Fig. 4b) even when the proviral loads did not decrease (Fig. 4c), again suggesting that STLV-1 infected clones that are prone to express sTax are selectively suppressed by immune responses to sTax and SBZ.

To confirm these findings, we analyzed whether CADM1 is expressed on STLV-1 infected cells. CADM1 is reported to be expressed on both ATL cells and HTLV-1 infected cells, suggesting that CADM1 is a good marker of HTLV-1 infection. Likewise, we found that STLV-1 infected monkey lymphoid cell line (Si2) also express CADM1 on their surfaces (Supplementary Fig. S4). When we analyzed CADM1 positivity among CD4^+^ T cells in treated JMs, we found that the number of CADM1^+^ infected cells, like the proviral load, was not decreased (Fig. 4d).

### Treg cell suppression by mogamulizumab does not confer abnormal activation of immune cells

**Figure 5:**
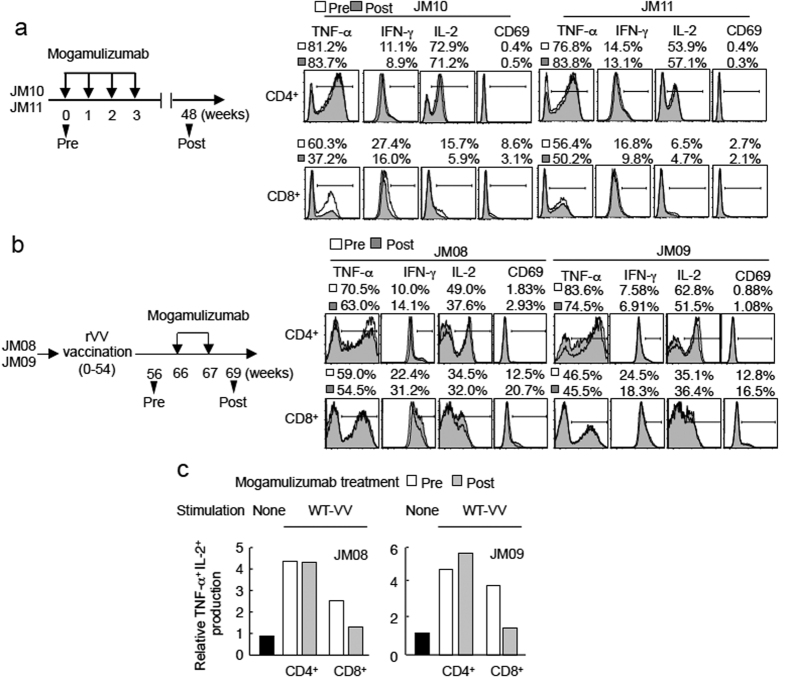
Immune responses after mogamulizumab treatment. (**a**) Two JMs were treated with mogamulizumab four times, and 48 weeks later, cells expressing various markers were measured. (**b,c**) Two monkeys were first vaccinated with rVV-sTax M22 or SBZ LL/AA and then treated twice with mogamulizumab. Two weeks after mogamulizumab treatment, cytokine production induced by PMA/ionomycin and CD69 expression were measured (**b**). The T-cell response (TNF-α and IL-2 production) to vaccinia virus was analyzed by stimulating with WT-VV-infected auto-PBMCs (**c**). Cytokine production in response to VV-uninfected PBMCs is shown as a control.

Does mogamulizumab enhance the host immune response to STLV-1 in a non-specific or specific manner? We evaluated whether mogamulizumab augments cytokine production and induces the activated phenotype of T cells *in vivo*. We found that cytokine production levels in mogamulizumab-treated monkeys were similar at 0 and 48 weeks after treatment (Fig. 5a). Likewise, cytokine production was not enhanced in monkeys 2 weeks after mogamulizumab treatment, although the number of CD69^+^ T cells increased slightly (Fig. 5b). To evaluate the effect of mogamulizumab on the immune response to specific antigens, we chose vaccinia virus antigen, since this virus can induce strong immune responses by infection through skin scarification^19^ and VV was used as a vector in this study. When PBMCs from vaccinated monkeys (JM08 and JM09) were stimulated by wild type VV-infected auto PBMCs, effector cytokine production (both IL-2 and TNF-α in CD4^+^ T cells was not enhanced by mogamulizumab treatment (Fig. 5c). In CD8^+^ T cells, the production of these cytokines was in fact lower after the antibody treatment (Fig. 5c). Thus mogamulizumab treatment appears to efficiently enhance the immune response to STLV-1 and possibly chronically infected pathogens by antigenic stimulation and suppressed effector Treg cells.

### Mogamulizumab treatment enhances HTLV-1-specific T-cell responses in some ATL patients

**Figure 6:**
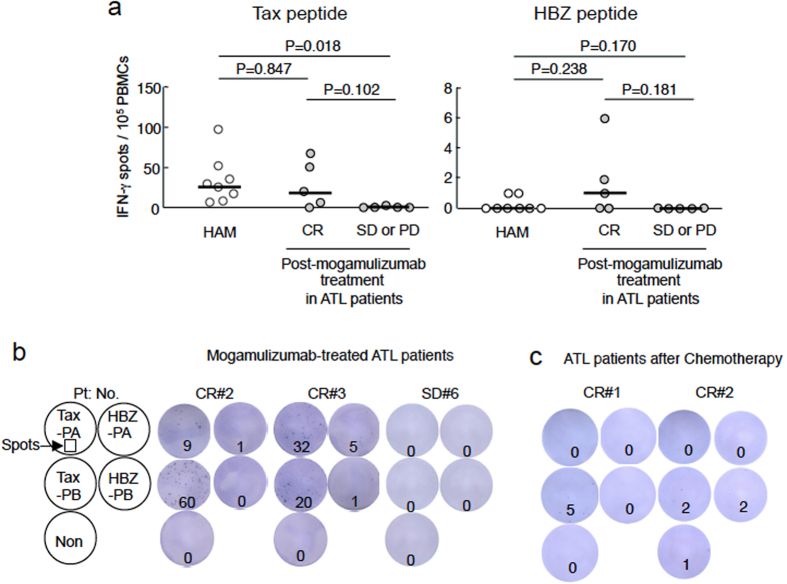
Enhanced T cell response to Tax and HBZ in mogamulizumab-treated ATL patients. (**a**) IFN-γ production in T cells from HAM/TSP (n = 8) and mogamulizumab-treated ATL patients (n = 10, 5 in complete remission (CR) and 5 in stable disease (SD) or progressive disease (PD)) was measured after stimulation with Tax and HBZ pooled peptides. Data shown are the total number of spots obtained by stimulation with Tax-PA (Tax_1–164_) and -PB (Tax_151–353_), or HBZ-PA (HBZ_1–104_) and -PB (HBZ_91–206_). (**b**) Two ATL patients (CR2 and CR3) showed enhanced HTLV-1 specific T-cell responses to Tax and HBZ after mogamulizumab treatment. (**c**) T-cell responses to Tax and HBZ in two ATL patients who were in CR after chemotherapy. Median values are represented by horizontal bar.

Results obtained from monkey experiments suggest that mogamulizumab specifically enhances immune responses to STLV-1, thus suppressing proviral load *in vivo*. To determine if the same is true in humans, we investigated T-cell responses to Tax and HBZ in ATL patients treated with mogamulizumab. The clinical description of these patients is shown in Supplementary Table S5. As controls, we measured memory T cell responses in eight HTLV-1 associated myelopathy/tropical spastic paraparesis (HAM/TSP) patients. High frequencies of anti-Tax CTLs have been reported in HAM/TSP than other HTLV-1 infected patients^20^. For HAM/TSP patients, the median number of IFN-γ spots in response to Tax and HBZ peptide stimulation was 27 and 0, respectively. In five ATL patients in complete remission (CR) who received mogamulizumab treatment, the spots were detected in four cases for Tax and three cases for HBZ (Fig. 6, Supplementary Table S6). In particular, strong immune responses were detected in two CR patients (ATL patients CR2 and CR3). On the other hand, spots were not observed in four ATL cases (ATL patients SD6 - PD10) in stable disease (SD) or progressive disease (PD). IFN-γ spots responding to Tax and HBZ were more prevalent in ATL patients in CR compared with patients in SD or PD, although the increases were not statistically significant (Fig. 6a,b). Furthermore, we analyzed two ATL cases in CR after chemotherapy. The number of spots was low in these patients (Fig. 6c). These data suggest that enhanced immune responses to HTLV-1 triggered by mogamulizumab are implicated in the control of ATL.

## Discussion

In this study, we showed that mogamulizumab activated anti-viral immunity, probably by enhancing the phagocytosis of infected cells and suppressing effector Treg cells. In cancer immunotherapy using mAbs, the clinical effect of mAbs is mainly mediated by ADCC and Fc-dependent phagocytosis against cancer cells directly^21^. It has been reported that DCs engulf antibody treated cancer cells via Fc-dependent phagocytosis, thereby stimulating the generation of cancer antigen-specific T cells^22^,^23^. Recently, anti-tumor monoclonal antibody has been shown to induce long-term anti-tumor cellular memory responses via stimulation of dendritic cells^24^. This study suggests that mogamulizumab-mediated increased engulfment of CCR4^+^ infected T cells by macrophages enhances antigen-presenting activity *in vivo* and potentiates T-cell responses to viral antigens. Thus, mogamulizumab can function both as a specific anti-cancer antibody and also as an enhancer of the immune response. This bimodal effect enables long-term suppression of virus-infected cells and ATL cells.

Loss of Treg cells *in vivo* is associated with a severe immune activated phenotype of leukocytes (especially T cells) in which peripheral tolerance is disrupted^25^. It has been reported that only effector Treg cells are targeted and suppressed by mogamulizumab treatment^15^. One tends to assume that T-cell responses would be non-specifically activated after administration of mogamulizumab. However, severe non-specific T-cell activation was not observed in mogamulizumab-treated monkeys (Fig. 5a,b). Mogamulizumab does not affect naïve Treg cells *in vivo* since they are CCR4 negative (Fig. 1d). Therefore, the remaining naïve Treg cells are implicated in controlling the immune system. It has been reported that depletion of effector Treg cells by mogamulizumab enhances T-cell responses to a cancer/testis antigen^15^. Our results suggest that simultaneous suppression of effector Treg cells and antigen stimulation can enhance the immune response to STLV-1 and HTLV-1. It has been reported that the frequency of CD4^+^Foxp3^+^ T cells was inversely correlated with the lytic activity of HTLV-1-specific CTLs in patients with ATL^26^, which is consistent with hypothesis that suppressed Treg cells are linked with enhanced T-cell responses. Mogamulizumab can do just that: deplete effector Treg cells while simultaneously enhancing the presentation of STLV-1 antigens *in vivo*.

In this study, we used STLV-1 infected JMs as models of HTLV-1 infection. Many animal models have been devised for HTLV-1 study^27^. Transgenic animals are powerful for analyzing the functions of viral genes *in vivo*. However, it is impossible to study the immune response to HTLV-1 in these transgenic animals. Humanized mice are also useful models, although the immune responses to HTLV-1 are dampened. HTLV-1 can infect a variety of animals including rabbit, rat, mouse, and monkey. The merit of HTLV-1 infected animals is that immune responses to HTLV-1 can be analyzed in these models. In particular, the macaque model is beneficial due to its similarities to humans^28^. Furthermore, when we use naturally STLV-1 infected macaques, we can choose macaques with a high proviral load. This is useful for observing the effect of vaccination on proviral load.

It is noteworthy that, after vaccination of JMs, the number of sTax-expressing T cells decreased while proviral load did not change much. Similarly, in unvaccinated monkeys treated with mogamuzilumab, the proviral load rebounded after 10 weeks, but the number of sTax-expressing T cells remained low. Thus it appears that STLV-1 infected clones that do not express sTax (or produce very small amounts of sTax) expanded after the immune response was enhanced by vaccination or mogamuzilumab. This finding suggests that STLV-1 infected cells are heterogeneous with regard to viral gene expression. It has been reported that Tax-expressing cells are significantly more frequent in clones of low abundance *in vivo*^29^, and less frequent in clones of high abundance. These data suggest that CTLs against Tax influence the clonality of infected cells. It has also been reported that Tax-expressing HTLV-1 infected cells are associated with the pathogenesis of HAM/TSP via the production of IFN-γ^30^, indicating that infected cells that are prone to express Tax might be more pathogenic in HTLV-1 associated diseases. Thus vaccination against Tax might be beneficial for the prevention and treatment of HTLV-1 associated diseases, even if such vaccination does not decrease overall proviral load.

As shown in this study, the suppressive effect of mogamulizumab on Treg cells persisted for a longer time than the antibody itself persisted *in vivo*. This apparent puzzle is understood when one considers the fact that most HTLV-1 (or STLV-1) infected cells are Foxp3^+^ T cells^31^. This happens in STLV-1 infected JM (Supplementary Fig. S5). Since mogamulizumab activates a specific immune response against STLV-1 infected cells, the population of Foxp3^+^ STLV-1 infected T cells will remain decreased for a longer time than mogamuzilumab persists.

The recent development of humanized antibodies to immune checkpoints has allowed the exploration of new strategies in cancer immunotherapy. Mogamuzilumab can reduce the number effector Treg cells, and it is well known that Treg cells inhibit the T cell memory response against cancer, pathogens and vaccines *in vivo*^32^,^33^,^34^. But in addition, as observed in this study, mogamulizumab can induce long-term specific immune responses to STLV-1. This study suggests that mogamulizumab exerts anti-ATL effects through two pathways: direct killing by ADCC, and enhanced T-cell responses to HTLV-1 via suppression of Treg cells and augmentation of APC activity. Mogamulizumab treatment that targets ATL cells by ADCC and enhanced T-cell responses is a novel potential therapy for ATL and HTLV-1 associated diseases.

## Methods

### Animals and human samples

All experiments using animals and human samples were performed in accordance with guidelines and regulations approved by Kyoto University. All animal experiments using mice and monkeys were approved by Kyoto University (approval numbers D12-02, D13-02, D14-02, R12-01, R13-01 and R14-01). C57BL/6 mice were purchased from CLEA Japan (Tokyo). All mice (6–14 weeks of age) used in this study were maintained in an SPF facility for breeding of mice. Experimental vaccination of JMs and mice was done in biosafety level-2 and 3 facilities, and the animals were handled according to protocols approved by Kyoto University. Blood samples were collected from ATL and HAM/TSP patients and peripheral blood mononuclear cells (PBMC) were isolated by Ficoll-Paque Plus (GE Healthcare Bio-Sciences) density gradient centrifugation. This study was conducted according to the principles in the Declaration of Helsinki, and approved by the Institutional Ethics Committee of Kyoto University (approval number E1649). Written informed consent was obtained from all ATL and HAM/TSP patients.

### Reagents, cells, and promoter assay

Reagents, cell culture, and the promoter assay are described in Supplementary Methods.

### Enzyme-linked immunosorbent spot (ELISPOT) assay

CD4^+^ or CD8^+^ T cells were isolated from mouse splenocytes and monkey PBMCs using magnetic particles (BD Bioscience). The ELISPOT assay was carried out using the mouse or human IFN-γ ELISPOT kit (MABTECH) according to the manufacturer’s protocol. Mouse splenocytes (5 × 10^5^ cells) or monkey PBMCs (10^5^ cells) were seeded into the ELISPOT plates and stimulated with 1 μM pooled peptide (four sTax peptide pools (P1: sTax_1–98_, P2: sTax_85–164_, P3: sTax_151–266_ and P4: sTax_253–353_) or four SBZ peptide pools (P1: SBZ_1–62_, P2: SBZ_49–104_, P3: SBZ_91–152_ and P4: SBZ_139–209_)) in the presence of 1 μg/ml of anti-CD28 antibody for 6 hours. IFN-γ spots were developed using the AP Conjugate Substrate Kit (Biorad) and measured by an ImmunoSpot S6 Analyzer (CTL). The specific T cell response was calculated as specific IFN-γ production (IFN-γ spots) = peptide-stimulated wells – non-stimulated wells. The specific T cell response of CD4^+^ and CD8^+^ cells was measured in CD8-depleted and CD4-depleted cells, respectively.

Sequences of peptides used in this study are summarized in Tables S1 and S2.

### Real-time polymerase chain reaction assay for proviral load

Proviral load was measured by real-time PCR quantifying the copy number of tax and RAG1 as previously described^17^. DNA samples from monkey PBMCs were subjected to quantification of STLV-1 proviral load using the StepOnePlus™ real time PCR system and TaqMan Gene Expression Master Mix (Applied Biosystems). Primer and probe sequences are described below; for monkey RAG1 as an internal control, 5′-CCCACCTTGGGACTCAGTTCT-3′ (sense), 5′-CACCCGGAACAGCTTAAATTTC-3′ (anti-sense) and 5′-CCCCAGATGAAATTCAGCACCCATATA-3′ (probe) were used. For STLV-1 Tax, 5′-CTACCCTATTCCAGCCCACTAG-3′ (sense), 5′-CGTGCCATCGGTAAATGTCC-3′ (anti-sense) and 5′-CACCCGCCACGCTGACAGCCTGGCAA-3′ (probe) were used.

### *Ex vivo* culture of monkey PBMCs in the presence of mogamulizumab

To measure antibody-dependent phagocytosis triggered by mogamulizumab, we differentiated monkey macrophages from PBMCs using human macrophage colony-stimulating factor (R&D systems) and human IL-1β (Miltenyi Biotec). Target CD4^+^ T cells were enriched from the PBMCs of an STLV-1 infected monkey, stained with PKH26 (Sigma-Aldrich), and treated with 5 μg/ml mogamulizumab in PBS for 20 min at room temperature. 2.5 × 10^4^ macrophages were co-cultured with 2.5 × 10^5^ target CD4^+^ T cells for 2 hours. Target cells engulfed by macrophages were measured as PKH26^+^CD11b^+^ Target cells engulfed by macrophagescells using flow cytometry.

To analyze CCR4^+^ Target cells engulfed by macrophagescells after treatment by mogamulizumab, we seeded CD8 depleted PBMCs (from unvaccinated and untreated monkeys) at 10^5^ cells per well in a round-bottom 96-well plate and treated them with 0–10 μg/ml mogamulizumab for 5 days. After treatment, CCR4 expression on CD4^+^ Target cells engulfed by macrophagesT cells was measured by flow cytometry.

For cytokine production assays, 1–2 × 10^6^ PBMCs from unvaccinated JM08 and JM09 monkeys were pre-cultured for 6 hours. Then all cells were harvested and re-seeded in culture medium supplemented with 10 μg/ml mogamulizumab or isotype control. IL-2 and IL-7 were added at 100 U/ml and 40 ng/ml, respectively. The medium was changed twice a week. After 11–18 days, living cells were stimulated with auto-PBMCs that had been pulsed with 1 μM pooled peptides (sTax PA: sTax_1–164_, PB: sTax_151–353_ and SBZ PA: SBZ_1–104_, PB: SBZ_91–206_) for 6 hours and labeled with cell tracer dye. Cytokine production in the tracer negative cell population was measured by flow cytometry.

### Generation of recombinant vaccinia viruses (rVV) and vaccination

All rVVs used in this experiment were generated as previously reported^35^. In brief, rVV was generated by homologous recombination in chicken embryonic fibroblasts. An antigen gene was inserted into the hemagglutinin gene of the LC16m8 strain. sTax M22 and SBZ LL/AA were used as antigens. The rVVs generated were cloned by adsorption with chicken red blood cells on RK13 cells. Purified rVVs were propagated and titrated on the RK13 cell line and stored at −80 °C.

Expression of the gene inserted in rVV was checked by immunoblotting or reverse transcription PCR (RT-PCR). Anti-Tax antibody (MI73) was used to detect sTax^36^. RT-PCR was done using the following primer sets: SBZ 5′-GGGCCGTTTCGATGTTTACCTGTTCCC-3′ (sense) and 5′-GCAGTCTCCCCTGCCAATAGTTAACCTC-3′ (anti-sense), rabbit β-actin 5′-GGCCCGACTCGTCATACTCCTGCTTGC-3′ (sense) and 5′-CATGAAGTGCGACGTGGACATCCGC-3′ (anti-sense).

The skin of mice (C57BL/6) or monkeys was shaved for vaccination and 10 μl (10^7^ PFU) of rVV was inoculated by skin scarification using a bifurcated needle. In mouse experiments, booster vaccination was repeated 5 times, once every 3 weeks beginning 4 weeks after initial vaccination. One week after the last inoculation, splenocytes were harvested and stored in liquid nitrogen until assay. In monkey experiments, booster vaccination was repeated every 4 weeks. PBMCs from monkeys were collected every 2 weeks. For induction of effective immunization in mice and monkeys, we changed scarification sites at every inoculation.

### Mogamulizumab treatment and vaccination in infected Japanese macaques (JMs)

STLV-1-infected Japanese macaques JM08 (16 years old), JM09 (14 years old), JM10 (15 years old) and JM11 (14 years old) were used for rVV vaccination and mogamulizumab treatment. (Mf-6 and Mf-7 in our previous study were renamed JM10 and JM11, respectively^17^.) STLV-1-infected JMs were screened using the Serodia HTLV-1 kit (Fuji Rebio), and provirus load was measured by real-time PCR. Mogamulizumab treatment was carried out as reported in our previous study^17^. In brief, 1 mg/kg mogamulizumab was diluted in 40 ml saline and intravenously infused into each monkey over 20 minutes. rVV was inoculated as described above. sTax-positive cells in CD8-depleted PBMCs were detected by flow cytometry: cells were cultured *ex vivo* for 24 hours and stained with anti-Tax Ab (MI73), biotin-labeled anti-mouse IgG3, and streptavidin-conjugated PE. sTax expressing CD4 T cells were gated based on CD4 T cells from a non-Tax expressing cell line or monkey non-T cell population (CD4 and CD8 negative).

### The immune response before and after mogamulizumab treatment

For non-specific stimulation, PBMCs treated with phorbol myristate acetate (PMA) and ionomycin for 4 hours in presence of protein transport inhibitor. Cytokine production and cell surface markers were measured by flow cytometry. CD69 expression was measured in un-stimulated samples.

For evaluation of the memory response against vaccinia virus in vaccinated JM08 and JM09, PBMCs from these monkeys were collected before and after mogamulizumab treatment. 5–10 × 10^6^ of auto-PBMCs before vaccination were infected with WT-VV (MOI = 4) for 24 hours at 37 °C, labeled with tracer dye, and used as stimulator cells. 10^6^ stimulator cells were co-cultured with responder PBMCs in the presence of protein transport inhibitor in a 96-well round bottom plate. IL-2 and TNF-α production from CD4^+^ or CD8^+^ T cells in the tracer-dye negative population was detected using flow cytometry.

### High throughput sequencing of provirus integration sites

The provirus integration sites in STLV-1-infected cells of the JMs were analyzed by high throughput sequencing as previously described^17^. Clonality was calculated as reported^17^.

### The HTLV-1 specific T-cell response in mogamulizumab-treated ATL and HAM/TSP patients

Clinical information for these patients is presented in Table S5. PBMCs were separated from whole blood using Ficoll, and 10^5^ PBMCs were stimulated with pooled Tax peptides (Tax-PA: Tax_1–164_ and -PB: Tax_151–353_) or pooled HBZ peptides (HBZ-PA: HBZ_1–104_, and -PB: HBZ_91–206_) in the presence of 1 μg/ml anti-CD28 antibody for 6 hours. IFN-γ spots were developed using the AP Conjugate Substrate Kit (Biorad) and measured with the ImmunoSpot S6 Analyzer (CTL). All sequences of peptides used in this study are summarized in Tables S3 and S4.

### Statistical analysis

Multiple data comparisons for *in vitro* and *ex vivo* or survival experiments were performed using the Student’s unpaired *t*-test or logrank test, respectively.

## Additional Information

**How to cite this article**: Sugata, K. *et al*. Enhancement of anti-STLV-1/HTLV-1 immune responses through multimodal effects of anti-CCR4 antibody. *Sci. Rep*. **6**, 27150; doi: 10.1038/srep27150 (2016).

## Supplementary Material

Supplementary Information
