# Supplementary material for: Enhancement of anti-STLV-1/HTLV-1 immune responses through multimodal effects of anti-CCR4 antibody
Source: Sci Rep. 2016 Jun 2;6:27150. doi: 10.1038/srep27150 (PMC4890010; doi:10.1038/srep27150)
Supplement: Supplementary Information [file srep27150-s1.pdf]

Supplementary Information

# Enhancement of anti-STLV-1/HTLV-1 immune responses through multimodal effects of anti-CCR4 antibody

Kenji Sugata[1,2], Jun-ichirou Yasunaga[1], Michi Miura[1], Hirofumi Akari[3], Atae Utsunomiya[4], Kisato Nosaka[5], Yuko Watanabe[6], Hitoshi Suzushima[6], Ki-Ryang Koh[7], Masanori Nakagawa[8],Michinori Kohara[9], Masao Matsuoka[1]

[1]Laboratory of Virus Control, Institute for Virus Research, Kyoto University, Kyoto, Japan
[2]Japan Society for the Promotion of Science (JSPS), Chiyoda-ku, Tokyo, Japan [3]Laboratory of Evolutional Virology, Institute for Virus Research, Kyoto University, Kyoto, Japan
[4]Department of Hematology, Imamura Bun-in Hospital, Kagoshima, Japan
[5]Department of Hematology, Kumamoto University School of Medicine, Kumamoto, Japan
[6]Department of Hematology, Kumamoto Shinto General Hospital, Kumamoto, Japan
[7]Department of Hematology, Osaka General Hospital of West Japan Railway Company, Osaka, Japan
[8]Department of Neurology, Graduate School of Medical Science, Kyoto Prefectural University of Medicine, Kyoto, Japan
[9]Department of Microbiology and Cell Biology, Tokyo Metropolitan Institute of Medical Science, Tokyo, Japan

**Table S1**

Sequences of sTax overlap peptides used in this study.

| Pep No | Name | Start | End | Sequence | Hydro | MWt |
|---|---|---|---|---|---|---|
| 1 | sTax pep1 | 1 | 20 | MAHFPGFGQSLLYGYPVYVF | 0.69 | 2293.7 |
| 2 | sTax pep2 | 7 | 26 | FGQSLLYGYPVYVFGDCVQG | 0.58 | 2212.5 |
| 3 | sTax pep3 | 13 | 32 | YGYPVYVFGDCVQGDWCPIS | 0.61 | 2268.6 |
| 4 | sTax pep4 | 19 | 38 | VFGDCVQGDWCPISGGLCSA | 0.55 | 2014.3 |
| 5 | sTax pep5 | 25 | 44 | QGDWCPISGGLCSARLHRHA | 0.33 | 2164.5 |
| 6 | sTax pep6 | 31 | 50 | ISGGLCSARLHRHALLATCP | 0.45 | 2076.5 |
| 7 | sTax pep7 | 37 | 56 | SARLHRHALLATCPEHQITW | 0.39 | 2340.7 |
| 8 | sTax pep8 | 43 | 62 | HALLATCPEHQITWDPIDGR | 0.38 | 2273.6 |
| 9 | sTax pep9 | 49 | 68 | CPEHQITWDPIDGRVIGSAL | 0.41 | 2207.5 |
| 10 | sTax pep10 | 55 | 74 | TWDPIDGRVIGSALQFLIPR | 0.48 | 2254.6 |
| 11 | sTax pep11 | 61 | 80 | GRVIGSALQFLIPRLPSFPT | 0.55 | 2169.6 |
| 12 | sTax pep12 | 67 | 86 | ALQFLIPRLPSFPTQRTSKT | 0.38 | 2301.7 |
| 13 | sTax pep13 | 73 | 92 | PRLPSFPTQRTSKTLKVLTP | 0.27 | 2267.7 |
| 14 | sTax pep14 | 79 | 98 | PTQRTSKTLKVLTPPATHTT | 0.18 | 2178.5 |
| 15 | sTax pep15 | 85 | 104 | KTLKVLTPPATHTTPNIPPS | 0.34 | 2113.5 |
| 16 | sTax pep16 | 91 | 110 | TPPATHTTPNIPPSFFQAVR | 0.38 | 2179.5 |
| 17 | sTax pep17 | 97 | 116 | TTPNIPPSFFQAVRKYSPFR | 0.34 | 2353.7 |
| 18 | sTax pep18 | 103 | 122 | PSFFQAVRKYSPFRNGYMEP | 0.27 | 2421.8 |
| 19 | sTax pep19 | 109 | 128 | VRKYSPFRNGYMEPTLGQQL | 0.22 | 2384.8 |
| 20 | sTax pep20 | 115 | 134 | FRNGYMEPTLGQQLPTLSFP | 0.41 | 2296.6 |
| 21 | sTax pep21 | 121 | 140 | EPTLGQQLPTLSFPDPGLRP | 0.37 | 2163.5 |
| 22 | sTax pep22 | 127 | 146 | QLPTLSFPDPGLRPQNLYTL | 0.46 | 2270.6 |
| 23 | sTax pep23 | 133 | 152 | FPDPGLRPQNLYTLWGNSVV | 0.45 | 2273.6 |
| 24 | sTax pep24 | 139 | 158 | RPQNLYTLWGNSVVCMYLYQ | 0.54 | 2448.9 |
| 25 | sTax pep25 | 145 | 164 | TLWGNSVVCMYLYQLSPPIT | 0.71 | 2285.7 |
| 26 | sTax pep26 | 151 | 170 | VVCMYLYQLSPPITWPLLPH | 0.87 | 2370.9 |
| 27 | sTax pep27 | 157 | 176 | YQLSPPITWPLLPHVIFCHP | 0.84 | 2358.8 |
| 28 | sTax pep28 | 163 | 182 | ITWPLLPHVIFCHPGQLGAF | 0.83 | 2246.7 |
| 29 | sTax pep29 | 169 | 188 | PHVIFCHPGQLGAFLTNVPY | 0.64 | 2210.6 |
| 30 | sTax pep30 | 175 | 194 | HPGQLGAFLTNVPYKRMEEL | 0.30 | 2300.7 |
| 31 | sTax pep31 | 181 | 200 | AFLTNVPYKRMEELLYKIFL | 0.51 | 2489.0 |
| 32 | sTax pep32 | 187 | 206 | PYKRMEELLYKIFLNTGATI | 0.39 | 2400.9 |
| 33 | sTax pep33 | 193 | 212 | ELLYKIFLNTGATIILPEDC | 0.57 | 2266.7 |
| 34 | sTax pep34 | 199 | 218 | FLNTGATIILPEDCLPTTLF | 0.66 | 2179.6 |
| 35 | sTax pep35 | 205 | 224 | TIILPEDCLPTTLFQPTRAP | 0.54 | 2226.6 |
| 36 | sTax pep36 | 211 | 230 | DCLPTTLFQPTRAPATLTAW | 0.51 | 2203.6 |
| 37 | sTax pep37 | 217 | 236 | LFQPTRAPATLTAWQNGLLP | 0.50 | 2195.6 |
| 38 | sTax pep38 | 223 | 242 | APATLTAWQNGLLPFQSTLT | 0.52 | 2130.4 |
| 39 | sTax pep39 | 229 | 248 | AWQNGLLPFQSTLTTPGLIW | 0.66 | 2243.6 |
| 40 | sTax pep40 | 235 | 254 | LPFQSTLTTPGLIWTFTDGT | 0.59 | 2196.5 |
| 41 | sTax pep41 | 241 | 260 | LTTPGLIWTFTDGTPMVSGP | 0.57 | 2091.4 |
| 42 | sTax pep42 | 247 | 266 | IWTFTDGTPMVSGPCPRDGQ | 0.37 | 2165.4 |
| 43 | sTax pep43 | 253 | 272 | GTPMVSGPCPRDGQPSLVLQ | 0.35 | 2039.4 |
| 44 | sTax pep44 | 259 | 278 | GPCPRDGQPSLVLQSSSFIF | 0.43 | 2135.4 |
| 45 | sTax pep45 | 265 | 284 | GQPSLVLQSSSFIFHKFQTK | 0.38 | 2279.6 |
| 46 | sTax pep46 | 271 | 290 | LQSSSFIFHKFQTKAYHPSF | 0.40 | 2400.7 |
| 47 | sTax pep47 | 277 | 296 | IFHKFQTKAYHPSFLLSHGL | 0.49 | 2371.8 |
| 48 | sTax pep48 | 283 | 302 | TKAYHPSFLLSHGLIQYSSF | 0.49 | 2296.6 |
| 49 | sTax pep49 | 289 | 308 | SFLLSHGLIQYSSFHNLHLL | 0.65 | 2326.7 |
| 50 | sTax pep50 | 295 | 314 | GLIQYSSFHNLHLLFEEYTN | 0.46 | 2425.7 |
| 51 | sTax pep51 | 301 | 320 | SFHNLHLLFEEYTNIPISLL | 0.62 | 2400.8 |
| 52 | sTax pep52 | 307 | 326 | LLFEEYTNIPISLLFNKEEA | 0.44 | 2383.7 |
| 53 | sTax pep53 | 313 | 332 | TNIPISLLFNKEEANDTDHE | 0.11 | 2300.5 |
| 54 | sTax pep54 | 319 | 338 | LLFNKEEANDTDHEPQMLPG | 0.12 | 2298.5 |
| 55 | sTax pep55 | 325 | 344 | EANDTDHEPQMLPGGLKPPN | 0.06 | 2160.4 |
| 56 | sTax pep56 | 331 | 350 | HEPQMLPGGLKPPNEKHFRE | 0.09 | 2341.7 |
| 57 | sTax pep57 | 334 | 353 | QMLPGGLKPPNEKHFRETDV | 0.12 | 2293.6 |

**Table S2**
Sequences of SBZ overlap peptides and vaccinia peptides used in this study.

| Pep No | Name | Start | End | Sequence | Hydro | MWt |
|---|---|---|---|---|---|---|
| 1 | SBZ pep1 | 1 | 20 | MAASGPFRCLPVPCPEDLLV | 0.59 | 2115.6 |
| 2 | SBZ pep2 | 7 | 26 | FRCLPVPCPEDLLVDDLVDG | 0.50 | 2215.6 |
| 3 | SBZ pep3 | 13 | 32 | PCPEDLLVDDLVDGLLSLEE | 0.40 | 2184.5 |
| 4 | SBZ pep4 | 19 | 38 | LVDDLVDGLLSLEEDLNKQR | 0.17 | 2284.6 |
| 5 | SBZ pep5 | 25 | 44 | DGLLSLEEDLNKQRTEEESV | -0.05 | 2304.5 |
| 6 | SBZ pep6 | 31 | 50 | EEDLNKQRTEEESVLDGLLS | -0.05 | 2304.5 |
| 7 | SBZ pep7 | 37 | 56 | QRTEEESVLDGLLSLEEECY | 0.14 | 2342.5 |
| 8 | SBZ pep8 | 43 | 62 | SVLDGLLSLEEECYGQQQVP | 0.33 | 2207.5 |
| 9 | SBZ pep9 | 49 | 68 | LSLEEECYGQQQVPLREESP | 0.16 | 2334.6 |
| 10 | SBZ pep10 | 55 | 74 | CYGQQQVPLREESPPRGETY | 0.11 | 2337.6 |
| 11 | SBZ pep11 | 61 | 80 | VPLREESPPRGETYRDRQRR | -0.20 | 2497.8 |
| 12 | SBZ pep12 | 67 | 86 | SPPRGETYRDRQRRAEEKRK | -0.44 | 2515.8 |
| 13 | SBZ pep13 | 73 | 92 | TYRDRQRRAEEKRKRKRERE | -0.67 | 2747.1 |
| 14 | SBZ pep14 | 79 | 98 | RRAEEKRKRKREREKEEEEQ | -0.76 | 2700.0 |
| 15 | SBZ pep15 | 85 | 104 | RKRKREREKEEEEQIAEFLR | -0.40 | 2660.0 |
| 16 | SBZ pep16 | 91 | 110 | REKEEEEQIAEFLRKKEEKK | -0.39 | 2576.9 |
| 17 | SBZ pep17 | 97 | 116 | EQIAEFLRKKEEKKARRRRR | -0.39 | 2628.1 |
| 18 | SBZ pep18 | 103 | 122 | LRKKEEKKARRRRREEEKAA | -0.61 | 2568.0 |
| 19 | SBZ pep19 | 109 | 128 | KKARRRRREEEKAAYRARRK | -0.61 | 2615.1 |
| 20 | SBZ pep20 | 115 | 134 | RREEEKAAYRARRKREEEER | -0.61 | 2647.9 |
| 21 | SBZ pep21 | 121 | 140 | AAYRARRKREEEERLERKRR | -0.52 | 2659.0 |
| 22 | SBZ pep22 | 127 | 146 | RKREEEERLERKRRLAEQGA | -0.45 | 2539.9 |
| 23 | SBZ pep23 | 133 | 152 | ERLERKRRLAEQGAKRARQR | -0.40 | 2507.9 |
| 24 | SBZ pep24 | 139 | 158 | RRLAEQGAKRARQRDTRKEK | -0.47 | 2453.8 |
| 25 | SBZ pep25 | 145 | 164 | GAKRARQRDTRKEKIKELGV | -0.29 | 2339.7 |
| 26 | SBZ pep26 | 151 | 170 | QRDTRKEKIKELGVDGYARQ | -0.22 | 2390.7 |
| 27 | SBZ pep27 | 157 | 176 | EKIKELGVDGYARQLESEVD | -0.01 | 2278.5 |
| 28 | SBZ pep28 | 163 | 182 | GVDGYARQLESEVDSLEAER | -0.03 | 2223.3 |
| 29 | SBZ pep29 | 169 | 188 | RQLESEVDSLEAERKRLLQE | -0.09 | 2428.7 |
| 30 | SBZ pep30 | 175 | 194 | VDSLEAERKRLLQEKEDLMG | -0.03 | 2359.7 |
| 31 | SBZ pep31 | 181 | 200 | ERKRLLQEKEDLMGEVNYWQ | 0.03 | 2564.9 |
| 32 | SBZ pep32 | 187 | 206 | QEKEDLMGEVNYWQGRLQAM | 0.13 | 2425.7 |
| 33 | SBZ pep33 | 190 | 209 | EDLMGEVNYWQGRLQAMWSQ | 0.31 | 2441.7 |

| Pep No | Name | Start | End | Sequence | Hydro | MWt |
|---|---|---|---|---|---|---|
| 1 | VV B8R | 20 | 27 | TSYKFESV | 0.08 | 960.1 |
| 2 | VV L2R | 53 | 61 | VIYIFTVRL | 0.73 | 1123.4 |
| 3 | VV K3L | 6 | 15 | YSLPNAGDVI | 0.30 | 1048.2 |

**Table S3**

Sequences of Tax overlap peptides used in this study.

| Pep No | Name | Start | End | Sequence | Hydro | MWt |
|---|---|---|---|---|---|---|
| 1 | Tax pep1 | 1 | 20 | MAHFPGFGQSLLFGYPVYVF | 0.73 | 2277.7 |
| 2 | Tax pep2 | 7 | 26 | FGQSLLFGYPVYVFGDCVQG | 0.62 | 2196.5 |
| 3 | Tax pep3 | 13 | 32 | FGYPVYVFGDCVQGDWCPIS | 0.64 | 2252.6 |
| 4 | Tax pep4 | 19 | 38 | VFGDCVQGDWCPISGGLCSA | 0.55 | 2014.3 |
| 5 | Tax pep5 | 25 | 44 | QGDWCPISGGLCSARLHRHA | 0.33 | 2164.5 |
| 6 | Tax pep6 | 31 | 50 | ISGGLCSARLHRHALLATCP | 0.45 | 2076.5 |
| 7 | Tax pep7 | 37 | 56 | SARLHRHALLATCPEHQITW | 0.39 | 2340.7 |
| 8 | Tax pep8 | 43 | 62 | HALLATCPEHQITWDPIDGR | 0.38 | 2273.6 |
| 9 | Tax pep9 | 49 | 68 | CPEHQITWDPIDGRVIGSAL | 0.41 | 2207.5 |
| 10 | Tax pep10 | 55 | 74 | TWDPIDGRVIGSALQFLIPR | 0.48 | 2254.6 |
| 11 | Tax pep11 | 61 | 80 | GRVIGSALQFLIPRLPSFPT | 0.55 | 2169.6 |
| 12 | Tax pep12 | 67 | 86 | ALQFLIPRLPSFPTQRTSKT | 0.38 | 2301.7 |
| 13 | Tax pep13 | 73 | 92 | PRLPSFPTQRTSKTLKVLTP | 0.27 | 2267.7 |
| 14 | Tax pep14 | 79 | 98 | PTQRTSKTLKVLTPPITHTT | 0.25 | 2220.6 |
| 15 | Tax pep15 | 85 | 104 | KTLKVLTPPITHTTPNIPPS | 0.40 | 2155.6 |
| 16 | Tax pep16 | 91 | 110 | TPPITHTTPNIPPSFLQAMR | 0.44 | 2219.6 |
| 17 | Tax pep17 | 97 | 116 | TTPNIPPSFLQAMRKYSPFR | 0.33 | 2351.8 |
| 18 | Tax pep18 | 103 | 122 | PSFLQAMRKYSPFRNGYMEP | 0.26 | 2419.8 |
| 19 | Tax pep19 | 109 | 128 | MRKYSPFRNGYMEPTLGQHL | 0.23 | 2425.8 |
| 20 | Tax pep20 | 115 | 134 | FRNGYMEPTLGQHLPTLSFP | 0.43 | 2305.7 |
| 21 | Tax pep21 | 121 | 140 | EPTLGQHLPTLSFPDPGLRP | 0.38 | 2172.5 |
| 22 | Tax pep22 | 127 | 146 | HLPTLSFPDPGLRPQNLYTL | 0.47 | 2279.6 |
| 23 | Tax pep23 | 133 | 152 | FPDPGLRPQNLYTLWGGSVV | 0.48 | 2216.5 |
| 24 | Tax pep24 | 139 | 158 | RPQNLYTLWGGSVVCMYLYQ | 0.57 | 2391.8 |
| 25 | Tax pep25 | 145 | 164 | TLWGGSVVCMYLYQLSPPIT | 0.74 | 2228.7 |
| 26 | Tax pep26 | 151 | 170 | VVCMYLYQLSPPITWPLLPH | 0.87 | 2370.9 |
| 27 | Tax pep27 | 157 | 176 | YQLSPPITWPLLPHVIFCHP | 0.84 | 2358.8 |
| 28 | Tax pep28 | 163 | 182 | ITWPLLPHVIFCHPGQLGAF | 0.83 | 2246.7 |
| 29 | Tax pep29 | 169 | 188 | PHVIFCHPGQLGAFLTNVPY | 0.64 | 2210.6 |
| 30 | Tax pep30 | 175 | 194 | HPGQLGAFLTNVPYKRIEEL | 0.33 | 2282.6 |
| 31 | Tax pep31 | 181 | 200 | AFLTNVPYKRIEELLYKISL | 0.45 | 2410.9 |
| 32 | Tax pep32 | 187 | 206 | PYKRIEELLYKISLTTGALI | 0.44 | 2321.8 |
| 33 | Tax pep33 | 193 | 212 | ELLYKISLTTGALIILPEDC | 0.60 | 2205.7 |
| 34 | Tax pep34 | 199 | 218 | SLTTGALIILPEDCLPTTLF | 0.68 | 2118.5 |
| 35 | Tax pep35 | 205 | 224 | LIILPEDCLPTTLFQPARAP | 0.61 | 2208.7 |
| 36 | Tax pep36 | 211 | 230 | DCLPTTLFQPARAPVTLTAW | 0.56 | 2201.6 |
| 37 | Tax pep37 | 217 | 236 | LFQPARAPVTLTAWQNGLLP | 0.54 | 2193.6 |
| 38 | Tax pep38 | 223 | 242 | APVTLTAWQNGLLPFHSTLT | 0.58 | 2167.5 |
| 39 | Tax pep39 | 229 | 248 | AWQNGLLPFHSTLTTPGLIW | 0.68 | 2252.6 |
| 40 | Tax pep40 | 235 | 254 | LPFHSTLTTPGLIWTFTDGT | 0.60 | 2205.5 |
| 41 | Tax pep41 | 241 | 260 | LTTPGLIWTFTDGTPMISGP | 0.60 | 2105.4 |
| 42 | Tax pep42 | 247 | 266 | IWTFTDGTPMISGPCPKDGQ | 0.40 | 2151.5 |
| 43 | Tax pep43 | 253 | 272 | GTPMISGPCPKDGQPSLVLQ | 0.38 | 2025.4 |
| 44 | Tax pep44 | 259 | 278 | GPCPKDGQPSLVLQSSSFIF | 0.44 | 2107.4 |
| 45 | Tax pep45 | 265 | 284 | GQPSLVLQSSSFIFHKFQTK | 0.38 | 2279.6 |
| 46 | Tax pep46 | 271 | 290 | LQSSSFIFHKFQTKAYHPSF | 0.40 | 2400.7 |
| 47 | Tax pep47 | 277 | 296 | IFHKFQTKAYHPSFLLSHGL | 0.49 | 2371.8 |
| 48 | Tax pep48 | 283 | 302 | TKAYHPSFLLSHGLIQYSSF | 0.49 | 2296.6 |
| 49 | Tax pep49 | 289 | 308 | SFLLSHGLIQYSSFHNLHLL | 0.65 | 2326.7 |
| 50 | Tax pep50 | 295 | 314 | GLIQYSSFHNLHLLFEEYTN | 0.46 | 2425.7 |
| 51 | Tax pep51 | 301 | 320 | SFHNLHLLFEEYTNIPISLL | 0.62 | 2400.8 |
| 52 | Tax pep52 | 307 | 326 | LLFEEYTNIPISLLFNEKEA | 0.44 | 2383.7 |
| 53 | Tax pep53 | 313 | 332 | TNIPISLLFNEKEADDNDHE | 0.06 | 2314.5 |
| 54 | Tax pep54 | 319 | 338 | LLFNEKEADDNDHEPQISPG | 0.02 | 2268.4 |
| 55 | Tax pep55 | 325 | 344 | EADDNDHEPQISPGGLEPPS | 0.00 | 2104.1 |
| 56 | Tax pep56 | 331 | 350 | HEPQISPGGLEPPSEKHFRE | 0.08 | 2271.5 |
| 57 | Tax pep57 | 334 | 353 | QISPGGLEPPSEKHFRETEV | 0.11 | 2237.5 |

**Table S4**

Sequences of HBZ overlap peptides used in this study.

| Pep No | Name | Start | End | Sequence | Hydro | MWt |
|---|---|---|---|---|---|---|
| 1 | HBZ pep1 | 1 | 20 | MAASGLFRCLPVPCPEDLLV | 0.63 | 2131.6 |
| 2 | HBZ pep2 | 7 | 26 | FRCLPVPCPEDLLVEELVDG | 0.51 | 2243.6 |
| 3 | HBZ pep3 | 13 | 32 | PCPEDLLVEELVDGLLSLEE | 0.29 | 2128.3 |
| 4 | HBZ pep4 | 19 | 38 | LVEELVDGLLSLEEELKDKE | 0.03 | 2216.4 |
| 5 | HBZ pep5 | 25 | 44 | DGLLSLEEELKDKEEEEAVL | -0.12 | 2204.3 |
| 6 | HBZ pep6 | 31 | 50 | EEELKDKEEEEAVLDGLLSL | 0.01 | 2288.5 |
| 7 | HBZ pep7 | 37 | 56 | KEEEEAVLDGLLSLEEESRG | -0.03 | 2232.4 |
| 8 | HBZ pep8 | 43 | 62 | VLDGLLSLEEESRGRLRRGP | 0.09 | 2252.6 |
| 9 | HBZ pep9 | 49 | 68 | SLEEESRGRLRRGPPGEKAP | -0.16 | 2221.5 |
| 10 | HBZ pep10 | 55 | 74 | RGRLRRGPPGEKAPPRGETH | -0.17 | 2224.5 |
| 11 | HBZ pep11 | 61 | 80 | GPPGEKAPPRGETHRDRQRR | -0.30 | 2297.5 |
| 12 | HBZ pep12 | 67 | 86 | APPRGETHRDRQRRAEEKRK | -0.47 | 2473.8 |
| 13 | HBZ pep13 | 73 | 92 | THRDRQRRAEEKRKRKKERE | -0.71 | 2693.0 |
| 14 | HBZ pep14 | 79 | 98 | RRAEEKRKRKKEREKEEEKQ | -0.78 | 2671.0 |
| 15 | HBZ pep15 | 85 | 104 | RKRKKEREKEEEKQIAEYLK | -0.46 | 2619.0 |
| 16 | HBZ pep16 | 91 | 110 | REKEEEKQIAEYLKRKEEEK | -0.42 | 2592.9 |
| 17 | HBZ pep17 | 97 | 116 | KQIAEYLKRKEEEKARRRRR | -0.43 | 2644.1 |
| 18 | HBZ pep18 | 103 | 122 | LKRKEEEKARRRRRAEKKAA | -0.56 | 2510.0 |
| 19 | HBZ pep19 | 109 | 128 | EKARRRRRAEKKAADVARRK | -0.55 | 2451.9 |
| 20 | HBZ pep20 | 115 | 134 | RRAEKKAADVARRKQEEQER | -0.50 | 2454.7 |
| 21 | HBZ pep21 | 121 | 140 | AADVARRKQEEQERRERKWR | -0.42 | 2597.9 |
| 22 | HBZ pep22 | 127 | 146 | RKQEEQERRERKWRQGAEKA | -0.49 | 2598.9 |
| 23 | HBZ pep23 | 133 | 152 | ERRERKWRQGAEKAKQHSAR | -0.40 | 2507.8 |
| 24 | HBZ pep24 | 139 | 158 | WRQGAEKAKQHSARKEKMQE | -0.26 | 2426.8 |
| 25 | HBZ pep25 | 145 | 164 | KAKQHSARKEKMQELGIDGY | -0.13 | 2317.7 |
| 26 | HBZ pep26 | 151 | 170 | ARKEKMQELGIDGYTRQLEG | -0.04 | 2322.6 |
| 27 | HBZ pep27 | 157 | 176 | QELGIDGYTRQLEGEVESLE | 0.10 | 2265.4 |
| 28 | HBZ pep28 | 163 | 182 | GYTRQLEGEVESLEAERRKL | -0.06 | 2363.6 |
| 29 | HBZ pep29 | 169 | 188 | EGEVESLEAERRKLLQEKED | -0.21 | 2387.6 |
| 30 | HBZ pep30 | 175 | 194 | LEAERRKLLQEKEDLMGEVN | -0.05 | 2400.7 |
| 31 | HBZ pep31 | 181 | 200 | KLLQEKEDLMGEVNYWQGRL | 0.18 | 2449.8 |
| 32 | HBZ pep32 | 187 | 206 | EDLMGEVNYWQGRLEAMWLQ | 0.37 | 2468.8 |

**Table S5**

Clinical information of mogamulizumab-treated ATL patients

| Patient ID | Disease status at analysis | Number of Mogamulizumab administration (times) | Time after Mogamulizumab treatment (weeks) |
|---|---|---|---|
| CR1 | CR | 5 | 87 |
| CR2 | CR | 4 | 13 |
| CR3 | CR | 8 | 44 |
| CR4 | CR | 6 | 173 |
| CR5 | CR | 5 | 56 |
| SD6 | SD | 3 | 11 |
| SD7 | SD | 3 | 7 |
| SD8 | SD | 8 | 9 |
| PD9 | PD | 1 | 3 |
| PD10 | PD | 1 | 26 |

**Table S6**

ELISPOT data in ATL and HAM/TSP patients.

| HAM | IFN-γ spots/$10^5$ PBMCs | | | IFN-γ spots/$10^5$ PBMCs | | |
|---|---|---|---|---|---|---|
| Pt. No. | Tax-PA | Tax-PB | Total | HBZ-PA | HBZ-PB | Total |
| HAM#1 | 4 | 20 | 24 | 1 | 0 | 1 |
| HAM#2 | 6 | 10 | 16 | 0 | 0 | 0 |
| HAM#3 | 3 | 4 | 7 | 0 | 0 | 0 |
| HAM#4 | 9 | 25 | 34 | 0 | 0 | 0 |
| HAM#5 | 92 | 3 | 95 | 0 | 0 | 0 |
| HAM#6 | 49 | 1 | 50 | 0 | 0 | 0 |
| HAM#7 | 1 | 4 | 5 | 0 | 0 | 0 |
| HAM#8 | 0 | 28 | 28 | 1 | 0 | 1 |

| Mogamulizumab-ATL | IFN-γ spots/$10^5$ PBMCs | | | IFN-γ spots/$10^5$ PBMCs | | |
|---|---|---|---|---|---|---|
| Pt. No. | Tax-PA | Tax-PB | Total | HBZ-PA | HBZ-PB | Total |
| CR#1 | 5 | 1 | 6 | 0 | 0 | 0 |
| CR#2 | 9 | 60 | 69 | 1 | 0 | 1 |
| CR#3 | 32 | 20 | 52 | 5 | 1 | 6 |
| CR#4 | 2 | 16 | 18 | 1 | 1 | 2 |
| CR#5 | 0 | 0 | 0 | 0 | 0 | 0 |
| SD#6 | 0 | 0 | 0 | 0 | 0 | 0 |
| SD#7 | 0 | 0 | 0 | 0 | 0 | 0 |
| SD#8 | 2 | 1 | 3 | 0 | 0 | 0 |
| PD#9 | 0 | 0 | 0 | 0 | 0 | 0 |
| PD#10 | 0 | 0 | 0 | 0 | 0 | 0 |

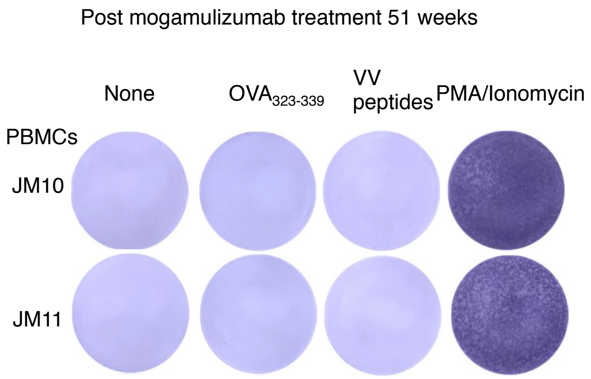


**Figure S1 Positive and negative controls in IFN-γ ELISPOT assay of monkey PBMCs.**

Monkey PBMCs were stimulated with $OVA_{323\text{-}339}$ and VV peptides (T cell epitopes in $H\text{-}2^b$ (C57BL/6) haplotypes) as negative control. We stimulated T cells by PMA and ionomycin as a positive control.

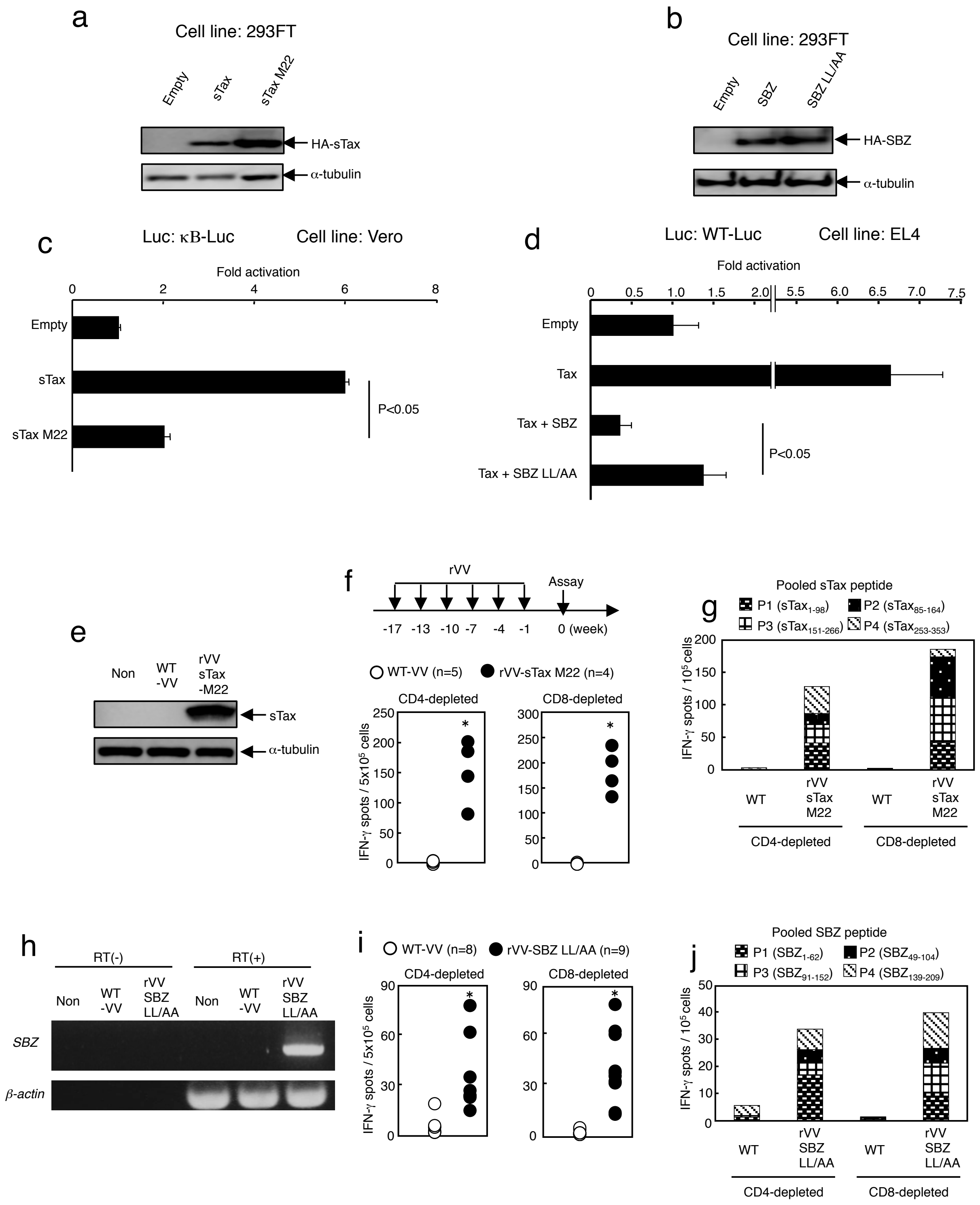
a
Cell line: 293FT
Empty
sTax
sTax M22
HA-sTax
α-tubulin
b
Cell line: 293FT
Empty
SBZ
SBZ LL/AA
HA-SBZ
α-tubulin
c
Luc: κB-Luc
Cell line: Vero
Fold activation
Empty
sTax
sTax M22
P<0.05
d
Luc: WT-Luc
Cell line: EL4
Fold activation
Empty
Tax
Tax + SBZ
Tax + SBZ LL/AA
P<0.05
e
Non
WT -VV
rVV sTax -M22
sTax
α-tubulin
f
rVV
Assay
-17 -13 -10 -7 -4 -1 0 (week)
WT-VV (n=5)
rVV-sTax M22 (n=4)
CD4-depleted
CD8-depleted
IFN-γ spots / 5x10^5 cells
g
Pooled sTax peptide
P1 (sTax1-98)
P2 (sTax85-164)
P3 (sTax151-266)
P4 (sTax253-353)
IFN-γ spots / 10^5 cells
WT
rVV sTax M22
CD4-depleted
CD8-depleted
h
RT(-)
RT(+)
Non
WT -VV
rVV SBZ LL/AA
SBZ
β-actin
i
WT-VV (n=8)
rVV-SBZ LL/AA (n=9)
CD4-depleted
CD8-depleted
IFN-γ spots / 5x10^5 cells
j
Pooled SBZ peptide
P1 (SBZ1-62)
P2 (SBZ49-104)
P3 (SBZ91-152)
P4 (SBZ139-209)
IFN-γ spots / 10^5 cells
WT
rVV SBZ LL/AA
CD4-depleted
CD8-depleted

**Figure S2 Generation of STLV-1 viral antigen-expressing vaccinia virus LC16m8 strain.**

**(a, b)** Point mutations in HTLV-1 Tax M22 (**a**) and HBZ LL/AA (**b**) were inserted into the equivalent sites in sTax and SBZ. 293FT cells were transfected with HA-tagged WT or mutant plasmid. After 48 hours of transfection, expression level was determined by western blot using rabbit polyclonal anti-HA antibody. Full-length data is presented in Supplementary Figure 1a and b. Those constructs expressed mutant viral antigen at a similar level to WT constructs. (**c, d)** The effect of viral proteins on transcriptional activity was impaired by the point mutations in sTax and SBZ. (**c)** Vero cells were transfected with kB-Luc and sTax or sTax M22 and the luciferase activity was measured. (**d)** EL4 cells were transfected with WT-Luc, containing Tax-inducible CREs of HTLV-1 LTR, Tax and SBZ or SBZ LL/AA. The cells were harvested after 48 hours. Relative luciferase activities were calculated as the ratio of firefly to *Renilla* luciferase activities. The bars represent the mean ± SD of triplicate experiments. (**e-j)** rVV vaccination induces an antigen-specific T-cell response in mice. sTax (**e**) and SBZ (**h**) expression from the generated rVV strains were confirmed by Western blot or RT-PCR in RK13 cells after 48 hours of post-infection, respectively. Expression of sTax or HBZ mutants was detected using anti-Tax antibody (MI73) or SBZ primer set, respectively. Full-length data is presented in Supplementary Figure 1c and d. rVV vaccination and detection of the specific immune response in mice were performed as shown in (**f**). CD4- or CD8-depleted splenocytes were stimulated with pooled overlapped peptides of sTax (**f**) or SBZ (**i**) and T-cell responses were measured by ELISPOT. The total numbers of spots from the four pools (P1-P4) are shown. (**g, j**) The proportion of T-cell responses induced by each of the four pooled-peptides of sTax (**g**) and SBZ (**j**) is shown. A representative result is shown from at least two similar experiments. *, $P<0.05$ by t-test.

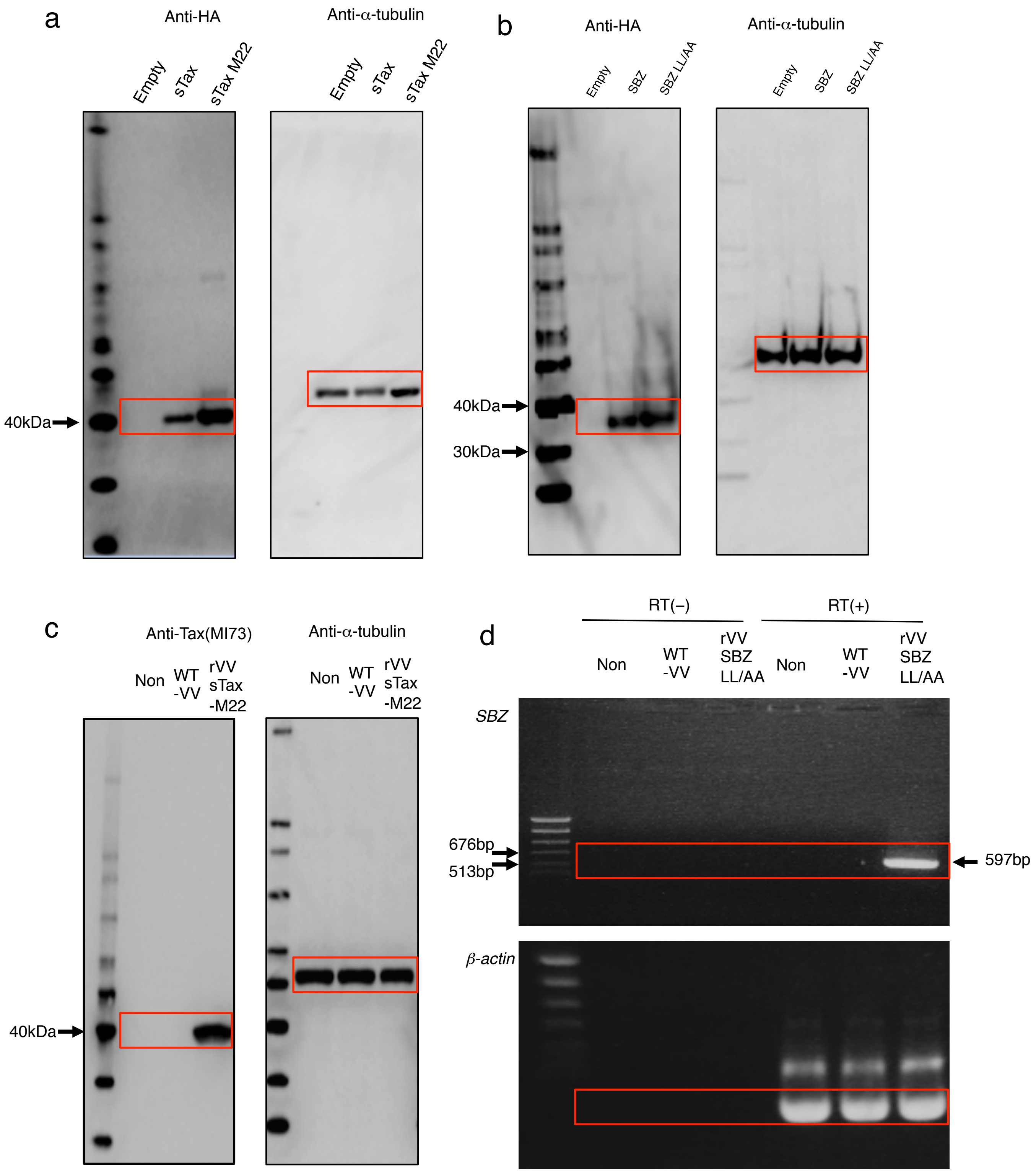


**Figure S3 Full-length data of Figure S1**

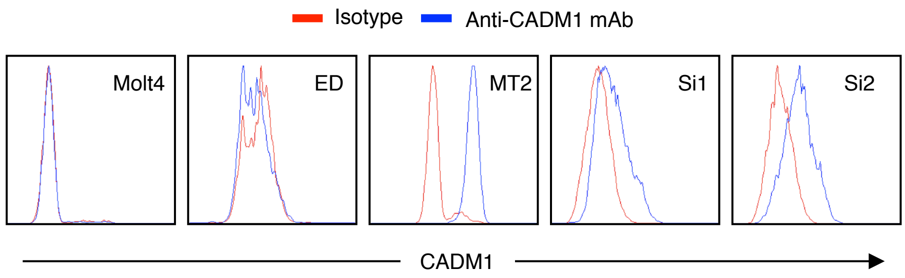


**Figure S4 STLV-1 infected cells express CADM1.**
We detected CADM1 expression using anti-human CADM1 mAb in cell lines. Si1 is a HTLV-1-immortalized monkey lymphocyte cell line. Si2 is a STLV-1-immortalized monkey lymphocyte cell line. Both T-cell lines derived from JMs express like a positive control, MT-2. ED and Molt4 are negative control for CADM1 expression.

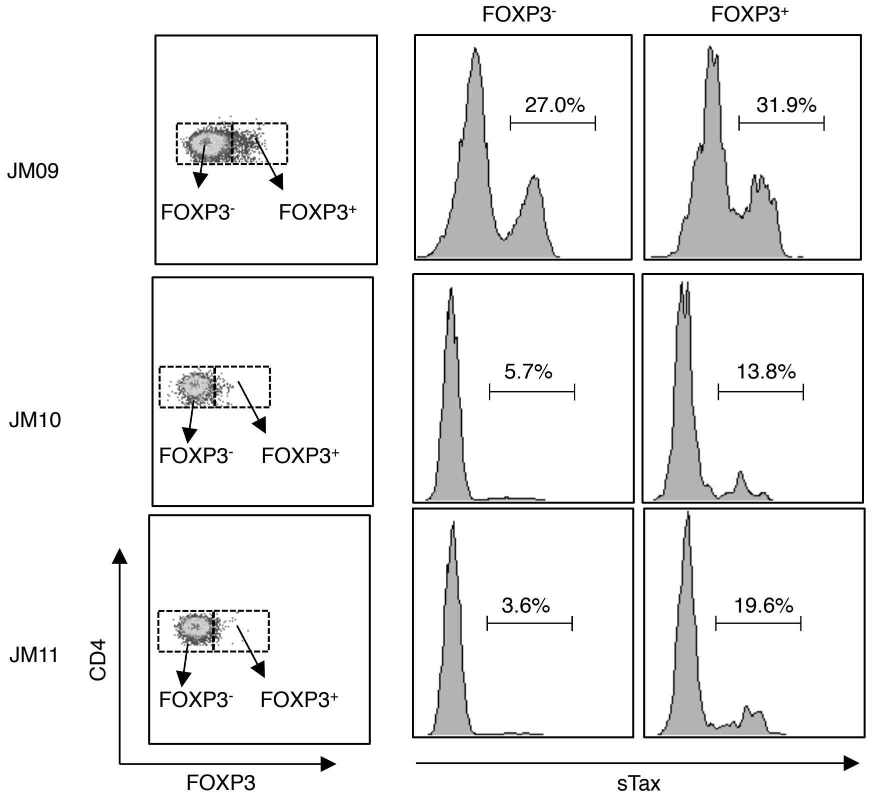


**Figure S5 sTax expression in CD4+ FOXP3+ cells .**

CD8-depleted monkey PBMCs were cultured for 24 hours. sTax expression was measured in $CD4^+$ $FOXP3^-$ or $CD4^+$ $FOXP3^+$ cells. Positive cells were gated based on each isotype control (>0.2%).

## Supplemental methods

**Antibodies and reagents**

The following antibodies were purchased from BD bioscience: purified mAbs to mouse IgG3 (R40-82) and CD28 (37.51) and human CD28 (28.2), CCR4 (1G1: recognizes a different epitope than mogamulizumab), IFN-g (4S.B3) and IL-2 (MQ1-17H12). MAbs to human CD4 (OKT-4), CD8a (RTP-T8), CD69 (FN50), and TNF-a (MAB11) were from Biolegend. MAbs to mouse CD11b (M1/70), human CD45RA (GRT22) and FOXP3 (PCH101) were from e-bioscience. Anti human CADM1 (3E1) was from MBL. Fluorescence-labeled anti-CCR4 antibody (clone KM2160, Kyowa Hakko Kirin Co. Ltd.), which is the original clone of mogamulizumab, was used for detection of $CCR4^{+}$ cells in monkey.

The following reagents were used for cell culture: protein transport inhibitor (BD PharMingen), streptavidin-PE (BD PharMingen), $OVA_{323-339}$(Bachem), tracer dye (CellTrace violet: Life Technologies) and human IL-2 (Wako). Mouse splenic T cells or monkey peripheral blood mononuclear cells (PBMCs) were depleted (<5%) or purified (>94%) using anti-mouse or anti-human CD4 or CD8 magnetic particles (BD Biosciences) according to the manufacturer's protocol. Whole overlap peptides (offset by 6 amino acids) were constructed based on the amino acid sequences of Tax, HBZ, sTax and SBZ (KURABO). All sequences of peptide used in this study are summarized in S1-S4 Table.

**Cells**

Suspension cell lines and primary cells were cultured in RPMI 1640 supplemented with 10% fetal bovine serum (FBS) and antibiotics. Si1 and Si2 cell lines were cultured in RPMI 1640 supplemented with 20% fetal bovine serum (FBS), antibiotics and IL-2. 2-Mercaptoethanol was added for culture of mouse cells. Vero, RK13 and chicken embryonic fibroblasts were cultured in Dulbecco's modified Eagle medium (DMEM) supplemented with 10% newborn calf serum, antibiotics and tryptose phosphate broth. PBMCs were separated from human or monkey whole blood using Ficoll. Flow cytometry was carried out using a FACS Verse with Suite Software or FACS CantoII with FACSDiva software (BD Pharmingen). Data was analyzed by FlowJo software (Treestar).

**Promoter assay**

The PathDetect pNF-kB-Luc Cis-Reporter Plasmid was purchased from Promega. WT-Luc (containing Tax-inducible CREs from HTLV-1 LTR) was a gift from J. Fujisawa (Kansai medical university). sTax (WT, M22 (T130A and L131S)) and SBZ (WT and LL/AA (L27A and L28A)) constructs were cloned into pCAG-HA. Vero ($10^5$) or EL4 ($2x10^5$ cells) were transiently co-transfected 24 hours after cell seeding using Lipofectamine™ LTX (Invitrogen) with 300 ng of reporter plasmid, 30 ng of *Renilla* luciferase control vector (pRL-TK), and 600 ng of the expression plasmid. Transfected Vero and EL4 were harvested 48 hours after transfection, respectively. Firefly and *Renilla* luciferase activities were then measured using the Pickagene kit (Toyo Inki). Relative luciferase activities were calculated as the ratio of firefly to *Renilla* luciferase activities.
